# Cardiometabolic, functional, and psychosocial effects of a remotely supervised home-based exercise program in individuals with type 2 diabetes (RED study): study protocol for a randomized clinical trial

**DOI:** 10.1186/s13063-023-07704-3

**Published:** 2023-10-19

**Authors:** Samara Nickel Rodrigues, Rodrigo Sudatti Delevatti, Mauricio Tatsch Ximenes Carvalho, Valentina Bullo, Marco Bergamin, Cristine Lima Alberton

**Affiliations:** 1 Federal University of Pelotas, Pelotas, Brazil; 2 Federal University of Santa Catarina, Florianópolis, Brazil; 3 Department of Medicine, University of Padova, Padua, Italy

**Keywords:** Diabetes mellitus, Exercise, Physical activity, Glycated hemoglobin, Randomized controlled trial

## Abstract

**Background:**

Type 2 diabetes mellitus (T2D) is a serious global health problem, and exercise is considered an essential non-pharmacological tool in T2D prevention and treatment. During periods of social isolation experienced by the COVID-19 pandemic, home-based exercise programs were strongly recommended as a strategy to facilitate exercise practice and reduce the negative impacts of social isolation. Remotely supervised exercise stands out as an easily accessible strategy after the pandemic, as it is a tool that aims to facilitate access to exercise by this population. The purpose of the RED study is to verify the effects of a remotely supervised home-based exercise program compared to a control group on cardiometabolic, functional, and psychosocial outcomes in patients with T2D.

**Methods:**

Participants are randomized into the control group (CG) and the intervention group (IG). Participants allocated to the CG receive recommendations for the practice of physical activity based on information from chapters of the Physical Activity Guide for the Brazilian Population, while the IG will perform a 12-week home-based exercise program supervised remotely by video call. The intervention has a weekly frequency of two sessions per week on non-consecutive days during the first 6 weeks and three sessions per week on non-consecutive days for the remaining 6 weeks. The RED study has HbA1c as the primary outcome, and the participants’ cardiometabolic, functional, and psychosocial parameters are assessed at baseline (week 0) and post-intervention (week 13).

**Discussion:**

Expected results of the proposed study will provide the knowledge base of health professionals and deliver more evidence for a growing area, i.e., home-based exercise and T2D. Additionally, this protocol aims to verify and demonstrate whether this program can be accessible and effective for different health outcomes in patients with T2D.

**Trial registration:**

The RED study protocol was prospectively registered at ClinicalTrials.gov (NCT05362071). Date registered April 6, 2022. https://clinicaltrials.gov/ct2/show/NCT05362071.

**Supplementary Information:**

The online version contains supplementary material available at 10.1186/s13063-023-07704-3.

## Background

Type 2 diabetes mellitus (T2D) is a metabolic disorder characterized by decreased insulin secretion and sensitivity and corresponds to 90% of all cases of diabetes [1]. Currently, just over half a billion people live with diabetes worldwide [2], with an estimate that by 2045, 783 million adults aged between 20 and 79 will be living with diabetes [1]. The diabetic population generally presents multimorbidity [3] and specific micro and macrovascular complications [4]. These complications increase the mortality risk, impairing the quality of life [4], and are associated with a greater likelihood of developing other physical and mental diseases. The treatment of T2D consists of medication use, diet, and physical exercise [5].

For disease control, glycated hemoglobin (HbA1c) is considered the gold standard marker, as it is a chronic measure effective for monitoring glycemic stability long term [6]. The American Diabetes Association (ADA) recommends the determination of HbA1c in patients with diabetes mellitus therapy in order to monitor the glycometabolic state and thus reduce the risk of the disease [5]. HbA1c predicts the risk of developing diabetic complications [7], and a reduction in levels of this marker is associated with a decreased risk of developing complications of the disease [8, 9]. In addition, high HbA1c values increase the risk of dyslipidemia [10, 11] and are also able to predict the development of arterial hypertension [12]. Moreover, there is an 18% increase in the risk of cardiovascular disease for every 1% increase in absolute HbA1c levels [9, 13]. In this context, exercise is considered an essential non-pharmacological tool in preventing and treating T2D, positively impacting HbA1c levels and different health parameters in this population [14–18].

Currently, Brazil is the 6th country in terms of incidence of diabetes in the world, with 15.7 million adults (20–79 years old) with the disease and with an estimated increase in the number of cases to 23.2 million cases in 2045. In this scenario, it is observed that the country has been presenting an increasing number of diagnoses compared to a decade ago, in which, in 2013, it had 11.9 million cases; in addition, it was responsible for 107,760 deaths in 2019, standing out as a serious public health problem [1, 19–21]. Additionally, the COVID-19 pandemic brought an increase in sedentary behavior and a decrease in physical activity levels [22], one of the pillars in the control and prevention of type 2 diabetes [5]. Faced with this scenario in which it is necessary to seek alternative ways to promote health, the practice of exercises through home exercise programs can become an alternative that is easily accessible and beneficial to this population.

In March 2020, the World Health Organization (WHO) declared the COVID-19 pandemic, which has been considered an unprecedented global health emergency. In this context, social distancing measures were recommended to slow the transmission of the virus. Such measures were necessary strategies; however, they resulted in negative consequences for individuals with TD2. Among them, we can mention a reduction in physical activity levels [23], worsening of glycemic [24] and metabolic control [25], impaired quality of life [26], increases in stress and anxiety levels [27], aggravated depressive symptoms [28], and a worsening of sleep quality [29]. In this context, it was necessary to look for alternatives to control T2D and the negative impacts of social isolation, highlighting the use of telehealth programs [30].

Noteworthy, it has to be highlighted that even before the pandemic, several studies corroborated the effects of home-based exercise programs using or supporting technology in individuals with T2D [31, 32], but those protocols were developed without supervision. Literature showed only [33] that verified effects of an exercise program remotely supervised by video conference in this population. Additionally, during periods of social isolation experienced by the COVID-19 pandemic, home-based exercise programs were strongly recommended as a useful strategy to facilitate the practice of exercises [34, 35]. Furthermore, the use of remote interventions will gain an even greater role in the health area, extending to the application of exercise interventions [36, 37]. However, literature is still scarce regarding studies verifying the results of remotely supervised home-based exercise programs in individuals with T2D. In this way, telehealth stands out as a strategy for the pandemic since it is a tool that facilitates or gives easy access to the practice of exercises. Therefore, we intend to evaluate the effects of a remotely supervised home-based exercise program compared with a control group on cardiometabolic, functional, and psychosocial results in patients with T2D.

## Methods

### Study design

The study is a clinical trial, randomized, single-center trial, and superiority trial with two parallel arms, blinded to outcome assessors and data analysts. The intervention consists of 12 weeks of a remotely supervised home-based exercise program in which cardiometabolic, functional, and psychosocial outcomes are analyzed in individuals with T2D. The RED study has a weekly frequency of two sessions per week on non-consecutive days during the first 6 weeks and three sessions per week on non-consecutive days for the remaining 6 weeks. This clinical trial was designed according to the guidelines for randomized clinical trials: Standard Protocol Items: Recommendations for Interventional Trials (SPIRIT) guidelines [38]. An additional file presents a SPIRIT checklist (see Additional File 1). This trial is being conducted at the Physical Education School of the Federal University of Pelotas (Brazil). The RED study protocol was prospectively registered at ClinicalTrials.gov (NCT05362071).

### Participants

The study participants comprise male and female patients with T2D from the city of Pelotas, located in the state of Rio Grande do Sul in the southern region of Brazil. Volunteers included in the sample are selected by recruitment advertisements on social media and basic health units (BHU) of five health districts located in the city. The BHU is the gateway to the Unified Health System (SUS) and offers multidisciplinary care for free. They are composed of Family Health teams that have a physician, nurse, nursing technician, community health agents, dentist, and oral health technician. In addition, the Family Health teams can work together with the support of the teams from the Expanded Centers for Family Health and Primary Care. These centers have professionals from other specialties, such as speech therapist, psychologist, occupational therapist, physiotherapist, pharmacist, nutritionist, and social worker, in accordance with the health demands of that territory.

### Eligibility criteria

Inclusion and exclusion criteria for participants are defined as follows.

### Inclusion criteria

- 1. Being under medical treatment using oral hypoglycemic agents;
- 2. Female and male patients with type 2 diabetes;
- 3. Aged ≥ 45 years old;
- 4. Not involved with physical exercises for at least three months (the regular practice of exercise was defined as performing any modality of physical training for at least 20 min on two or more days of the week);
- 5. Being semi-literate due to self-completion questionnaires.

### Exclusion criteria

- 1. Insulin prescription and use;
- 2. History of cardiovascular disease (except drug-controlled high blood pressure);
- 3. Presence of severe autonomic neuropathy, severe peripheral neuropathy or history of foot injuries, proliferative diabetic retinopathy, severe non-proliferative diabetic retinopathy;
- 4. Muscle or joint impairment that precludes performing physical exercises safely;
- 5. Lack of internet access.

### Recruitment

The recruitment period started in May 2022 and was completed in June 2023. Recruitment takes place through disseminating posters in BHU in the city and notes shared on social networks and the local newspaper. Then, patients are contacted by telephone, and complete information about the purpose of the study and a survey on the presence of conditions related to the inclusion criteria are provided. If the participant meets these criteria, he or she is invited to participate in the study.

### Randomization and allocation concealment

Once included in the study, the participant receives an internal number to be de-identified. The randomization sequence is stratified by sex and disease duration (< 5 years or ≥ 5 years) in blocks of different sizes with a 1:1 ratio in the Excel random function. The entire process of randomization and allocation into groups is carried out by an independent researcher not involved with the evaluations and intervention.

### Sample size

The sample calculation was performed using the GPower version 3.9.1.4 program for *F* tests (considering two groups and two measurements), adopting a significance level of *α* = 0.05 and 80% power. Data for sample size calculation were extracted from the study results [32] for the primary outcome of HbA1c (effect size *f* = 0.24), resulting in a total n of 38 subjects. Additionally, twelve individuals (≈ 30%) will be included in the study due to the possibility of sample losses, totaling 50 participants randomized into the two groups.

### Intervention and control procedure

Participants are randomly allocated to the intervention group (IG), which performs a remotely supervised home-based exercise program lasting a total of 12 weeks, or to the control group (CG). The CG receives general recommendations for physical activity. A detailed description of the intervention and control procedure is provided below:

### Remotely supervised exercise program

The remotely supervised home-based exercise program consists of 12 weeks. Sessions are held via video calls via *WhatsApp*, with a maximum of 5 participants per video call. The intervention has a weekly frequency of two sessions per week on non-consecutive days during the first 6 weeks and three sessions per week on non-consecutive days for the remaining 6 weeks. The 12-week exercise intervention consists of four mesocycles, each with 3-week duration. The session structure is maintained during each mesocycle and is changed for progress in the intensity or volume of its main part in the following mesocycle. The session structure consists of 5 min of warm-up, 37–57 min of the main part, in which a combined training program is carried out, and a final 5 min of stretching. Blocks 1 and 2 will consist of three strength exercises with body weight and alternative materials (500 ml plastic bottles with sand) and an aerobic exercise. Between blocks 1 and 2, and later in block 3, participants take a free walk with displacement in their homes’ available space. The aerobic training intensity is based on the Borg 6–20 Perception of Effort (RPE) Scale [39], while the resistance exercises are performed at the usual/fast speed of execution. The training progression is based on the increase in the number of sets, duration of effort, total duration of the session, intensity, frequency, and complexity of exercises. The alternative material (500 ml plastic bottles with sand) is delivered to all study participants on the day of the home visit. An additional file presents the full periodization of the study in more detail (see Additional File 2). Participants were instructed not to engage in any other type of activity involving exercise during the study period.

### Control group

Participants allocated to the CG receive recommendations for the practice of physical activity based on information from chapters of the Physical Activity Guide for the Brazilian Population (2021) [40]. When going through all the assessments carried out at baseline, the CG participants will receive through *WhatsApp* the information that is available in chapters 1, 4, and 5, which address the following topics: “Understanding Physical Activity,” “Physical Activity for Adults,” and “Physical Activity for the Elderly.” When not possible to send via *WhatsApp*, the print booklet is delivered to the participant. At the end of the 12 weeks, the same booklet is made available to the IG participants.

### Criteria for discontinuing study participation

Participants may be discontinued from the study due to withdrawal of participant consent, lack of interest, or willingness to continue. For participants allocated to the IG, participation is interrupted for safety reasons, such as medical advice or disease complication. In addition, muscle or joint injuries or a severe health event during the study precludes participation in intervention sessions.

### Strategies for trial retention

Participants allocated to the IG receive text messages to reinforce the date and time of interventions. We use *WhatsApp* messages to ask about adverse events if an IG participant misses a session. Sending messages is stopped for participants who declare their withdrawal from the study.

### Outcomes

Study results are assessed at baseline (week 0) and post-intervention (week 13). Outcomes are measured for all randomized participants, regardless of frequency or completion status. Participants who withdraw from the study at any time after randomization are invited to complete the final study assessments (12 weeks after the start of the intervention). Additionally, only the IG participants perform acute capillary blood glucose measurements before and immediately after an exercise session in the initial period of mesocycles 1, 2, and 4.

### Primary outcome

The primary outcome is the change in the HbA1c, evaluated through blood analysis. HbA1c was chosen as the primary outcome because of its importance in disease management. HbA1c is considered the gold standard in monitoring T2D, as it is a chronic measure and reflects the average blood glucose level over the previous 8 to 12 weeks [6]. Additionally, glycemic control, assessed by HbA1c levels, effectively reduces the risk of micro and macrovascular complications [41].

### Secondary outcomes

Clinically relevant secondary outcomes for individuals with T2D were established, including capillary blood glucose, systolic blood pressure (SBP), diastolic blood pressure (DBP), functional tests performance, depressive symptoms, diabetes-related emotional stress, sleep quality, and quality of life (QOL).

Capillary blood glucose is measured from blood samples collected from the participant’s fingertips. SBP and DBP are determined from office measurements. Capacity will be evaluated with the following tests: Arm Curl, 30 s-Chair Stand, Time Up and Go, 2 Minute Step Test, and Sit and Reach Flexibility Test. Depressive symptoms and diabetes-related emotional stress are measured using the Patient Health Questionnaire (PHQ-9) and the Brazilian version of the Problem Areas in Diabetes Scale (B-PAID). QOL and sleep quality will be measured using the EUROHIS-QOL 8-Item and Pittsburgh Sleep Quality Index Self-Report Questionnaire (PSQI).

### Other measures

Complementary measures of physical activity level, anthropometric data, eating habits, and subjective perception of well-being were assessed.

### Blinding

Blinding is applied to outcome assessors and data analysts of primary and secondary outcomes listed in this protocol. Due to the nature of the interventions, the team conducting the exercise sessions and the participants are not blinded. Participants are asked to withhold their assigned group and not talk about their interventions during outcome evaluations to ensure evaluator masking. In the event of unintentional uncovering for any reason, the researchers involved must notify the coordinator.

### Data collection

A single investigator carries out the home assessments pre- and post-intervention. During the assessments, he or she has a manual of standard operating procedures available. Two other researchers apply the online questionnaires. For this purpose, calls are made before to explain the procedures and immediately after the application to clarify doubts. In addition, the responsible investigator remains available to assist if any doubts arise during the completion of the questionnaire. All variables are assessed at baseline and after the intervention. Additionally, acute capillary blood glucose measurements are performed in the initial period of mesocycles 1, 2, and 4 and the subjective perception of well-being in week 13, both for the IG participants. The time scheme for study conduction is presented in Table 1.

**Table 1:** Time scheme for RED study conduction

Study period										
	**Screening**	**Baseline and allocation**		**Post-allocation**					**Close out**	
**Timepoint**	**T0**	**T1**	Allocation	**T2**	12 weeks				**T3**	**T4**
		Baseline		Intervention start					Intervention end	Final evaluation
					Mesocycle 1	Mesocycle 2	Mesocycle 3	Mesocycle 4		
**Enrolment:**										
Eligibility screen	X									
Informed consent	X									
Randomization			X							
**Interventions:**										
Intervention group				X	X	X	X	X	X	
Control group										
**Assessments:**										
***Primary outcomes***										
Glycated hemoglobin		X								X
***Secondary outcomes***		X								
Capillary blood glucose		X			X	X		X		X
Blood pressure		X								X
Functional capacity		X								X
Quality of Life Questionnaire		X								X
Depressive Symptoms Questionnaire		X								X
Sleep Quality Questionnaire		X								X
Emotional stress related to diabetes questionnaire		X								X
Subjective perception of well-being										X
**Other measures:**										
Anthropometric assessment		X								X
Physical activity levels		X								X
Eating habits		X								X

### Measurement of the primary outcome

#### Glycated hemoglobin

Blood samples are collected in a specialized laboratory for the HbA1c levels analysis of each participant. Whole blood samples (2 mL) are collected from the antecubital vein of participants after 8 h of fasting. HbA1c levels are determined using a high-performance liquid chromatography method by a private laboratory.

### Measurements of secondary outcomes

#### Capillary blood glucose

Initially, participants will rest for 5 min, sitting in a chair with both feet on the floor and the back resting on the back of a chair. Capillary blood glucose will be measured from blood samples (0.6 μL of blood) collected from the fingertips, using disposable lancets and reagent strips (Accu-Check Guide, São Paulo). After collection, blood samples will be immediately analyzed by a portable glucometer (Accu-Check Guide, Roche, São Paulo, Brazil).

### Blood pressure

Office SBP and DBP measurements are obtained using an oscillometric BP monitor (HEM-7320, OMRON, China). The participant is kept at rest for 5 min; then, a measurement is performed on each arm, and then two more measurements are obtained on the arm with the highest value, always with a 1-min interval between measurements.

### Functional tests

The 30-s Chair-Stand test measures the strength of the lower limbs. Participants are instructed to sit and stand up from a chair 42 cm high from the seat, without the aid of the upper limbs, as many times as possible for 30 s [42].

The Arm Curl test is performed to check upper body strength. The test is performed holding a dumbbell in the dominant hand, with a load corresponding to 2 kg for women and 4 kg for men. Participants are instructed to perform the maximum number of elbow flexion repetitions in the full range of motion for 30 s [42].

The Time Up and Go (TUG) is used to measure agility and dynamic balance. The test starts with the participant sitting in a chair with a cone positioned 3 m away in front of them. Each participant is instructed to get up from their chair, walk as quickly as possible without running, go around the cone, and return to the starting position. The shortest time of two attempts is the test result. In addition, the test is also performed at the usual walking speed [43].

The 2-min step test is carried out to estimate the aerobic capacity. The test measures the maximum number of knee raises the individual can perform in 2 min. At the indicative signal, the participant starts stationary gait (without running), completing the maximum knee raises within 2 min. The minimum knee height is a midpoint between the patella and the anterior superior iliac spine. The evaluator counts the number of right knee raises [42].

Sit and Reach Flexibility Test is applied using the Wells bench, which measures the lower limbs’ flexibility. Participants must be barefoot, sit facing the base of the box with their legs extended and together, place one of their hands on top of the other, and then raise their arms vertically. When the evaluator gives the signal, the participant bends the body forward and reaches with the fingertips as much as possible on the graduated ruler without bending the knees or using rocking movements (insistence). Two trials are performed, and the lowest value is recorded [44].

### Quality of life

QOL is measured using the EUROHIS-QOL 8-ITEM, an instrument validated for the Brazilian population [45]. The questionnaire consists of 8 items (general QOL, general health, energy, activity of daily living, self-esteem, social relationships, finances, and home). Each item is answered individually, using a Likert-type scale, from 1 to 5 points, ranging from “very bad to very good” (rating scale), “very dissatisfied to very satisfied” (satisfaction scale), and “not at all to extremely” (intensity scale). The total score ranges from 8 to 40 and indicates that the higher the score, the better the individual’s perception of their QOL.

### Depressive symptoms

Depressive symptoms are measured using the PHQ-9, an instrument validated for the Brazilian population [46]. It is a tool that aims to verify the presence of depressive symptoms in the last two weeks through a Likert-type scale from 0 to 3 points. It consists of nine questions with four response options ranging from “no, not one day” (0 points) to “almost every day” (3 points). The questionnaire also has the tenth question, referring to the interference of symptoms in daily life. In total, it is possible to have a score from 0 to 27, which indicates that the lower the score, the lower the depressive symptoms.

### Sleep quality

To measure sleep quality, the PSQI is used, an instrument validated for the Brazilian population [47]. The questionnaire includes 19 questions about the individual’s perception and five questions regarding the perception that the roommates of these individuals have about their sleep. If the participant does not have a roommate, the questions are not answered; therefore, they are not scored. These questions are grouped into seven components, with scores ranging from zero to three, where higher scores indicate worse sleep quality.

### Emotional stress related to diabetes

The B-PAID is used to analyze the emotional stress related to diabetes and the impact of diabetes and treatment on the lives of study participants. A 5-point Likert scale is used, ranging from “No problem = 0,” “Small problem = 1,” “Moderate problem = 2,” “Almost a serious problem = 3,” to “Serious problem = 4.” The B-PAID produces a total score that ranges from 0 to 100, where a high score indicates a high level of emotional distress. This total score is achieved by summing the 0–4 responses given on the 20 B-PAID items and multiplying this sum by 1.25 [48].

### Covariates

#### Anthropometric assessment

Body mass and height measurements are performed using a digital scale (HN-289, OMRON, China) and a compact stadiometer (MD, Brazil). From these data, the body mass index (BMI) will be calculated through the equation: BMI = body mass (kg)/height^2^ (m). Subsequently, the waist circumference is measured at the midpoint between the iliac crest and the last rib to verify the waist circumference/height ratio from these data***.***

### Physical activity levels

To measure the level of physical activity, the International Physical Activity Questionnaire—short version (IPAQ-C) is used in its short version, validated in its Brazilian version [49]. The IPAQ-C includes eight self-completion questions in different domains, such as work, leisure, domestic activities, and physical exercise. Data are expressed in minutes, and the metabolic equivalent is calculated (1 MET: 3.5 ml/kg/min). Like the other questionnaires, it is applied in electronic format, as already done in a previous study [50].

### Eating habits

The Food Frequency Questionnaire (FFQ) is used to control eating habits, as used in a previous study [51]. The questionnaire consists of a list of 16 foods prepared from previous studies [52, 53]. According to the Brazilian Food Guide Guidelines, these foods will be classified into two groups: in natura/minimally processed foods and processed or ultra-processed foods. Scores will be generated for these two food groups based on the frequency of consumption reported by the participants. The scores may vary from 0 to 32 points, in which higher scores represent better eating habits. A general healthy eating score will also be generated from the sum of all evaluated foods, which may vary from 0 to 64 points, where higher scores represent better eating habits.

### Adherence

For the IG participants, adherence measures are considered attendance and compliance in the intervention. Attendance is monitored through the online session’s frequency recording and treated as the percent of intervention sessions experienced by each participant, given the total number of applied sessions (thirty sessions). Compliance is treated as the percentage of intervention sessions performed without protocol deviations.

### Coordination and management of the trial

The coordinating center it is composed of the CLA, responsible for supervising all phases of the study as the trial stages progress. The trial steering committee is made up of RSD, MB, and VB and has the role of monitoring the study together with the coordinator center and supporting decisions. SNR is responsible for managing the study, recruiting, scheduling, logistically organizing home and laboratory evaluations, and carrying out home evaluations. MTXC does part of data management. In addition, we have employees responsible for implementing the training program.

### Data management

Data collected in online forms are identified by subject identification and contain instructions for standard operating procedures. A specific lead researcher performs the check for missing or inaccurate data. Furthermore, data collected during the course of the research will be kept strictly confidential and accessed only by members of the trial coordination team. The participant’s identity is preserved and identified by the identification number (ID), and their details will be stored in a database to which only the coordination team will have access. If requested by the research coordination team, the anonymous study data may be shared with other researchers.

### Statistical analyses

Descriptive statistics will be used to describe the sample or data set characteristics by mean, standard deviation, 95% confidence interval, and absolute and relative frequency. Data normality will be tested by the Shapiro–Wilk test, and variances homogeneity of the will be tested by the Levene test. Primary and secondary outcome analyses will be conducted using analysis of covariance (ANCOVA), wherein baseline values will be considered as covariates. The intention to treat (ITT) analysis will include all randomized participants, with multiple imputations applied for missing data. Furthermore, a per-protocol (PP) analysis will be performed, including only participants with a frequency higher than 70% in IG and both time points measurements in both groups. Effect sizes between-group will be computed based on Cohen’s *d* and the 95% confidence interval. For the IG, acute capillary blood glucose will also be compared pre- and post-session throughout the intervention, using mixed linear models (2 × 3; session and time factors), followed by post hoc Bonferroni test. All data will be analyzed using SPSS version 22 at an alpha level of 0.05.

### Data monitoring and auditing

The specific monitoring committee was not considered due to the nature of the study. However, the “trial steering committee” has the role of monitoring the study together with the coordinating center and supporting the decisions.

### Harms

The patients are informed that participation in the study involves a minimal probability of risks and discomforts, especially by the professional prescription and conduction of the exercise sessions. The common discomforts in exercise, such as fatigue during and post-exercise, are also explained to patients, which may require reducing the intensity or stopping the exercise. The research team is available to solve any adverse events. If adverse events are reported, they are collected and classified according to severity (i.e., mild, moderate, or severe), predictability (i.e., expected or unexpected), and potential relationship to study procedures (i.e., definitely related, possibly related, or unrelated).

Participants were instructed to take classes whenever possible with a companion at home. Additionally, the person responsible for applying the intervention has a list with the emergency contact telephone number and the address provided by the participants. In case of any more serious adverse event, the person responsible will contact the mobile emergency service, provide the address participant, and immediately inform the emergency contacts. In addition, the participants were instructed regarding care with food, hydration, clothing, and medication for the classes.

### Ethics and dissemination

#### Research ethics approval

Study procedures were approved by the Human Research Ethics Committee of the Physical Education School of Federal University of Pelotas (Brazil) (CAAE: 55,791,622.8.0000.5313). During the home visit, the researcher responsible for the evaluations informs the recruited participants about all stages of the study, including possible risks and benefits, and an informed consent form is signed if they agree to participate in the study. The identity of the participants is preserved and identified by the ID. No identifying images or other personal or clinical details of participants are presented here or will be presented in reports of the trial results.

### Protocol amendments

If necessary, amendments to the study protocol will be communicated to the Human Research Ethics Committee of the Physical Education School of the Federal University of Pelotas (Brazil). At the same time, our research team will also update the protocol of the clinical trial registry.

### Access to data

The datasets analyzed during the current study and statistical code will be available from the corresponding author on reasonable request, as is the full protocol, without violating participant confidentiality.

### Ancillary and post-trial care

After the RED study completion, the blood test results (weeks 0 and 13) will be delivered to the participants, in addition to an easy-to-understand report on the study results. In addition, timely information from tests performed can be provided to participants for treatment issues if requested.

### Dissemination policy

Upon completion of the study, our dissemination plan aims to disseminate the results found to as many interested parties as possible. First, participants will receive their reports with their measurements and interpretations in language adapted to the f the lay public’s understanding. All participants will also receive general guidance on type 2 diabetes, general health care, and physical activity. In addition, the results will be published in the press with the main findings on the topic aimed at the general public. Academic-scientific dissemination will be done through scientific articles submitted in journals and presentations at events.

### Trial status

This manuscript is based on the trial protocol dated December 2022. At the time of submission, patient recruitment has begun. Patient recruitment started on May 2022 and was completed on June 2023.

## Discussion

The trial intends to evaluate the effects of a remotely supervised home-based exercise program on cardiometabolic, functional, and psychosocial results in patients with T2D, and it is essential to highlight that the RED study has important characteristics. First, this population has a high prevalence; just over half a billion people live with diabetes worldwide, which means that more than 10.5% of the world’s adult population now has this condition [2]. T2D accounts for over 90% of all diabetes worldwide [1], and these patients have a higher risk of overall mortality than individuals without diabetes [54]. Given the current scenario of the disease, it is crucial to investigate the effectiveness of exercise interventions in important outcomes for controlling T2D. Additionally, this population has a low level of physical activity, which is related to different barriers that make it challenging to adhere to physical exercise [55, 56]. Thus, more favorable alternatives for exercise practice that confront these barriers and promote greater participation by these individuals should be studied and implemented.

During the COVID-19 pandemic, low levels of physical activity were observed in individuals with T2D due to the adoption of social distancing measures [23, 57]. Thus, considering the negative impact of physical inactivity on health, performing home-based exercises stood out as a fundamental alternative to mitigate physical inactivity. Additionally, new practices strengthened during the COVID-19 pandemic, such as telehealth (i.e., using technology for remote monitoring of patients with T2D) [30, 37], are also being adopted in the current scenario. However, the home-based exercise and T2D scenario still need greater scientific consistency and a greater number of intervention studies [58]. Therefore, it is essential to highlight the importance of carrying out this type of intervention that will be used in the RED, involving the performance of a remotely supervised home-based exercise training.

The exercise protocol performed in the RED study has original training characteristics. One of them is the combined training using aerobic exercises associated with strength exercises performed with the body weight and alternative materials. Interventions with combined aerobic and strength exercises may be superior to either mode alone [17]. Additionally, the exercise protocol has a progression concerning the number of sets, intensity, weekly frequency, duration of effort, total duration of the session, and complexity of the exercises. This detailing is a positive characteristic of our study since many studies fail to describe accurate information about exercise periodization and prescription.

Finally, the results of the proposed study are expected to benefit the knowledge base of health professionals and provide more evidence for a growing area, i.e., home-based exercise and T2D. Additionally, the expectation is to verify and demonstrate whether this program can be accessible and effective for different health outcomes in patients with T2D.

### Supplementary Information

**Additional file 1.**

**Additional file 2.**

## Abbreviations

ADA: American Diabetes Association
BHU: Basic health units
BMI: Body mass index
B-PAID: Brazilian version of the Problem Areas in Diabetes Scale
CG: Control group
DBP: Diastolic blood pressure
FFQ: Food Frequency Questionnaire
GEE: Generalized estimating equations
HbA1c: Glycated hemoglobin
ID: Identification number
IG: Intervention group
IPAQ-C: International Physical Activity Questionnaire—short version
ITT: Intention to treat
PHQ-9: Patient Health Questionnaire
PP: Per-protocol
PSQI: Pittsburgh Sleep Quality Index Self-report Questionnaire
QOL: Quality of life
RPE: Rating of perceived exertion
SBP: Systolic blood pressure
SUS: Unified Health System
T2D: Type 2 diabetes mellitus
TUG: Time Up and Go
WHO: World Health Organization
