# Supplementary material for: Cardiometabolic, functional, and psychosocial effects of a remotely supervised home-based exercise program in individuals with type 2 diabetes (RED study): study protocol for a randomized clinical trial
Source: Trials. 2023 Oct 19;24:679. doi: 10.1186/s13063-023-07704-3 (PMC10588211; doi:10.1186/s13063-023-07704-3)
Supplement: Supplementary file 2 — Additional file 2. [file 13063_2023_7704_MOESM2_ESM.pdf]

Box 1. Periodization of week 1 to 6 of the exercise program.

Training weeks: 1 to 3
Frequency: twice a week
Session Duration: 37 min

Warm-up:
Trunk articular mobility
Hip articular mobility
2 sets - 10 repetitions each

BLOCK 1

Exercise	Sets	Duration	Interval between exercises	Intensity	Interval between sets
Sit and stand-up<br>Wall push-up<br>Bilateral calf raise	2	30 s	30 s	Usual velocity	1 min
Stationary gait		1,5 min		RPE 11-13	

BLOCK 2

Exercise	Sets	Duration	Interval between exercises	Intensity	Interval between sets
Single arm dumbell row<br>Bridge<br>Abdominal crunch	2	30s	30s	Usual velocity	1 min
Stationary gait		1,5 min		RPE 11-13	

BLOCK 3

Exercise	Sets	Duration	Interval between exercises	Intensity	Interval between sets
Free walk	1	5 min	RPE 11-13		

Stretching exercises

Training weeks: 4 to 6
Frequency: twice a week
Session Duration: 53 min

Warm-up:
Shoulder articular mobility
Hip articular mobility
2 sets - 10 repetitions each

BLOCK 1

Exercise	Sets	Duration	Interval between exercises	Intensity	Intervalo between sets
Half squat<br>Unilateral row<br>Bilateral calf raise	3	30s	30s	Usual velocity	1 min
Stationary gait		1,5 min		RPE 11-13	
Free walk	1	5 min	RPE 11-13		

BLOCK 2

Exercise	Sets	Duration	Interval between exercises	Intensity	Intervalo between sets
Bridge<br>Floor press<br>Abdominal cross crunch	3	30s	30s	Usual velocity	1 min
Stationary gait		1,5 min		RPE 11-13	

BLOCK 3

Exercise	Sets	Duration	Interval between exercises	Intensity	Intervalo between sets
Free walk	1	5 min	RPE 11-13		

Stretching exercises

Box 2. Periodization of week 7 to 12 of the exercise program.

Training weeks: 7 to 9
Frequency: three times a week
Session duration: 53 min

Warm-up:
Shoulder articular mobility
Hip articular mobility
2 sets - 10 repetitions each

BLOCK 1

Exercise	Sets	Duration	Interval between exercises	Intensity	Interval between sets
Half squat<br>Unilateral row<br>Bilateral calf raise	3	30s	30s	Usual velocity	1 min
Stationary gait		1,5 min		RPE 11-13	
Free walk	1	5 min	RPE 11-13		
**BLOCK 2**					
Bridge<br>Floor press<br>Abdominal cross crunch	3	30s	30s	Usual velocity	1 min
Stationary gait		1,5 min		RPE 11-13	
**BLOCK 3**					
Free walk	1	5 min	RPE 11-13		

Stretching exercises

Training weeks: 7 to 9
Frequency: three times a week
Session duration: 57 min

Warm-up:
Shoulder articular mobility
Hip articular mobility
2 sets - 10 repetitions each

BLOCK 1

Exercise	Sets	Duration	Interval between exercises	Intensity	Interval between sets
Half squat<br>Bent-over two-arm dumbell row<br>Unilateral calf raise	3	20s	30s	Maximum execution speed	1 min
Stationary gait		2 min		RPE 13-15	
Free walk	1	5 min	RPE 13-15		
**BLOCK 2**					
Unilateral Bridge<br>Unilateral floor push up<br>Bird dog	3	20s	30s	Maximum execution speed	1 min
Stationary gait		2 min		RPE 13-15	
**BLOCK 3**					
Free walk	1	5 min	RPE 13-15		

Stretching exercises
